# The visual perception of emotion from masks

**DOI:** 10.1371/journal.pone.0227951

**Published:** 2020-01-15

**Authors:** J. Farley Norman, Sydney P. Wheeler

**Affiliations:** 1 Department of Psychological Sciences, Ogden College of Science and Engineering, Western Kentucky University, Bowling Green, Kentucky, United States of America; 2 Carol Martin Gatton Academy of Mathematics and Science, Bowling Green, Kentucky, United States of America; National Institutes of Health, UNITED STATES

## Abstract

Fifty-one adults evaluated visually-perceived emotions from 32 masks. These masks (held in the collection of the Kentucky Museum, located on the campus of Western Kentucky University) were created by artists from a wide variety of cultures spanning multiple continents. Each participant evaluated every mask along six dimensions: happiness, sadness, anger, fear, surprise, and disgust. No previous scientific study has ever studied the general effectiveness of masks (other than Japanese Noh masks) in producing perceptions of human emotion. The results showed that the masks were effective in producing substantial variations in perceived happiness, sadness, anger, fear, surprise, and disgust. The ability of the masks to produce effective perceptions of emotion was due to the artists’ inclusion of facial features that reliably signal emotions in everyday life.

## Introduction

Masks have been used for important religious, societal, and cultural purposes for at least ten thousand years [1–2]. As examples, consider the functions of masks in three world regions: Africa (e.g., Liberia), Southeast Asia (e.g., Borneo), and the Pacific Northwest coast of North America (e.g., British Columbia, Canada). In Africa, masked figures served as powerful agents of social control [3–5], exercising authority at judicial proceedings, settling disputes, serving as police, administering justice and discipline, creating laws, enforcing peace, etc. Masks were also used to promote the fertility of agricultural crops, and to facilitate the healing of illness. They were also employed at important personal events, such as birth, initiation into manhood, advancement in rank, and death. Finally, masks were used in comedy and entertainment. In Borneo [6], the functions of masks were similar to those employed in Africa: they were used to invoke protection for agricultural crops, to invoke protection from disease, were used at funerals and marriages, and for comedy. In the Pacific Northwest of North America [7], masks were utilized at judicial proceedings and were employed for crowd control and the maintenance of public order. Masks were also used at important social events from birth to death (e.g., birth, naming, marriage, illness, remembrances of dead family members).

**Fig 1:**
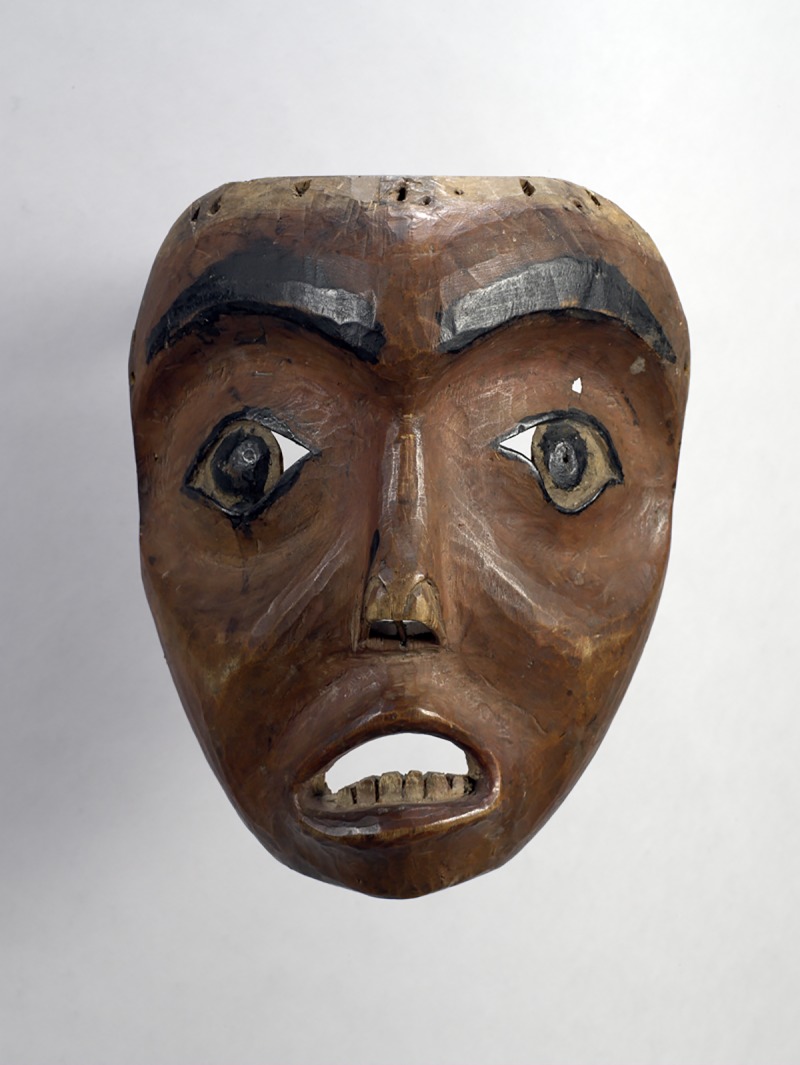
Sample mask. A photograph of a mask from the Pacific northwest coast of North America (this mask is possibly from the Tsimshian culture). This photograph of mask A4185 is reproduced courtesy of UBC Museum of Anthropology, Vancouver, Canada (the photographer was Jessica Bushey).

Many masks are created by the sculptor or artist with the deliberate intention of inducing particular emotion(s) in human perceivers [3, 7–9]. For example, according to Kecskési and Vajda [4] “some masks were intended to shock or horrify, others to astonish or to make audiences laugh” (p. 14). One can indeed perceive strong expressions of emotion from masks. Consider Fig 1, which shows a mask (A4185) held by the Museum of Anthropology at the University of British Columbia; when people view this mask, they usually perceive fear, sadness, surprise, or some combination of those emotions.

Given that artists have been creating masks for many thousands of years from cultures all around the world, it is surprising that almost no scientific research evaluating the perception of emotion from masks has been conducted to date. Some studies [10–11] have investigated the perception of emotion from Japanese Noh masks, but there is nothing else. In the study by Minoshita et al. [10], participants viewed images of a *single* Noh mask presented at a variety of orientations (face oriented up or down by various degrees) and were required to indicate, yes or no, for each image whether the mask appeared “sad”, “surprised”, or “happy”. The participants did not estimate the magnitude of the perceived emotion, but only made binary choices (yes or no) about whether the mask appeared to express a particular emotion. None of the other fundamental emotions (anger, fear, disgust) were evaluated at all. The results of this study (p. 89) indicated that the “perception of the emotion expressed by the image was shown to change with the inclination of the Noh mask in normal individuals”. In a subsequent similar study by Lyons and colleagues [11], participants also viewed images of a single Noh mask presented at a variety of orientations. These participants were also required to make a binary choice about each image, to indicate whether they perceived the depicted expression as “happy” or “sad”. Lyons et al. [11] concluded (p. 2243) that “faces tilted down have a happier cast than those tilted back”.

As will by now be clearly evident, no scientific investigation has ever thoroughly evaluated how emotions are perceived from masks; there is no literature concerning anything other than single Japanese Noh masks. This lack of information is surprising, because masks have played a primary and influential role in important societal events for many thousands of years by cultures all over the world (see beginning of introduction). The purpose of the current study was to rectify this lack of information and experimentally investigate emotion perception using a large number of masks obtained from many parts of the world (e.g., Bahamas, Bali, Brazil, Comoros Islands, Costa Rica, Ecuador, El Salvadore, Ghana, Greenland, Guatamala, Japan, Mexico, Paraguay, Senegal, Thailand, & Venezuela).

## Methods

### Apparatus and experimental stimuli

**Fig 2:**
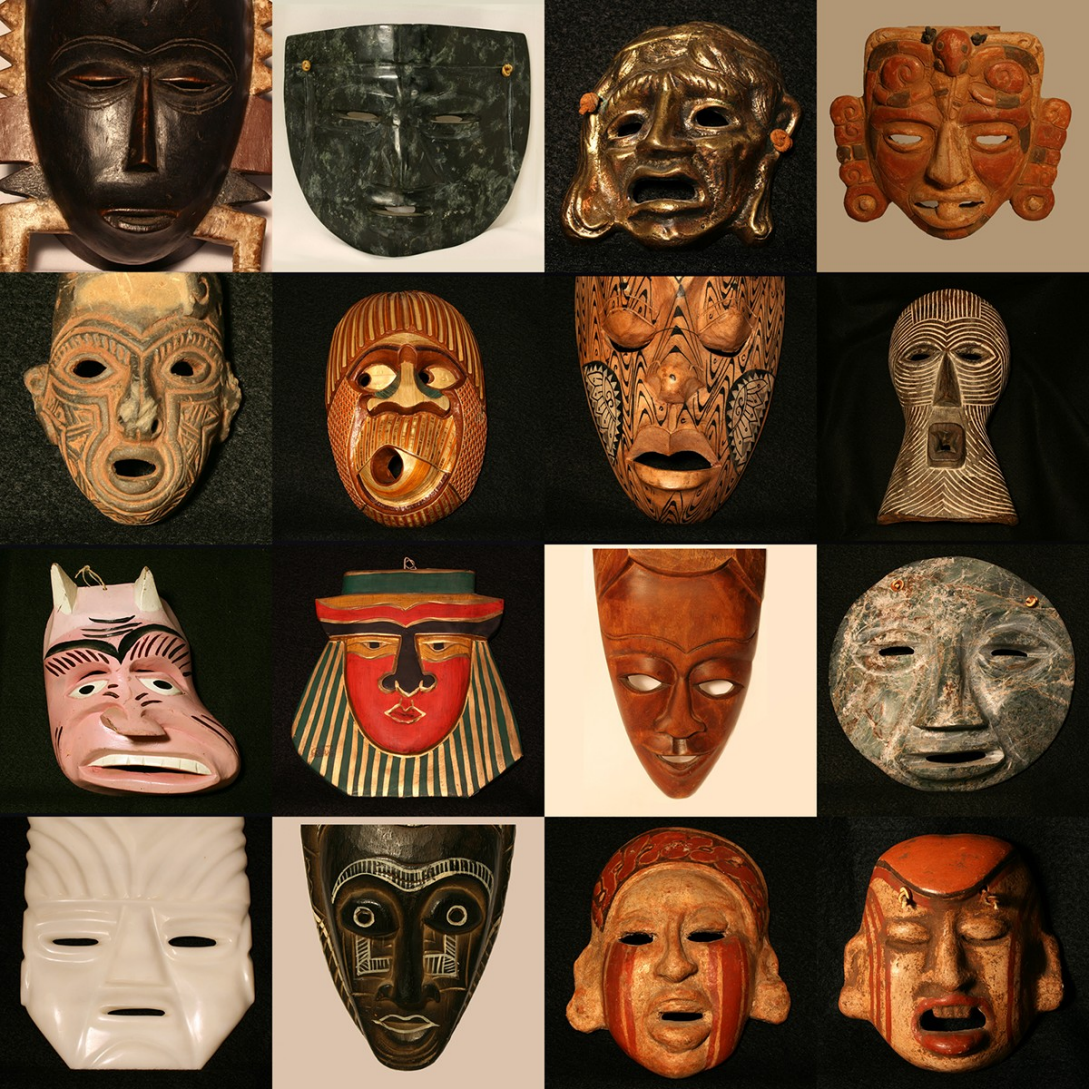
Photographs of masks 1–16 used in the current study. The masks are arranged numerically from top-left (Masks 1 & 2) to bottom-right (Masks 15 & 16). These photographs of a subset of the David Coffey collection are reproduced courtesy of the Kentucky Museum, located on the campus of Western Kentucky University (the photographer was the first author, J. Farley Norman).

**Fig 3:**
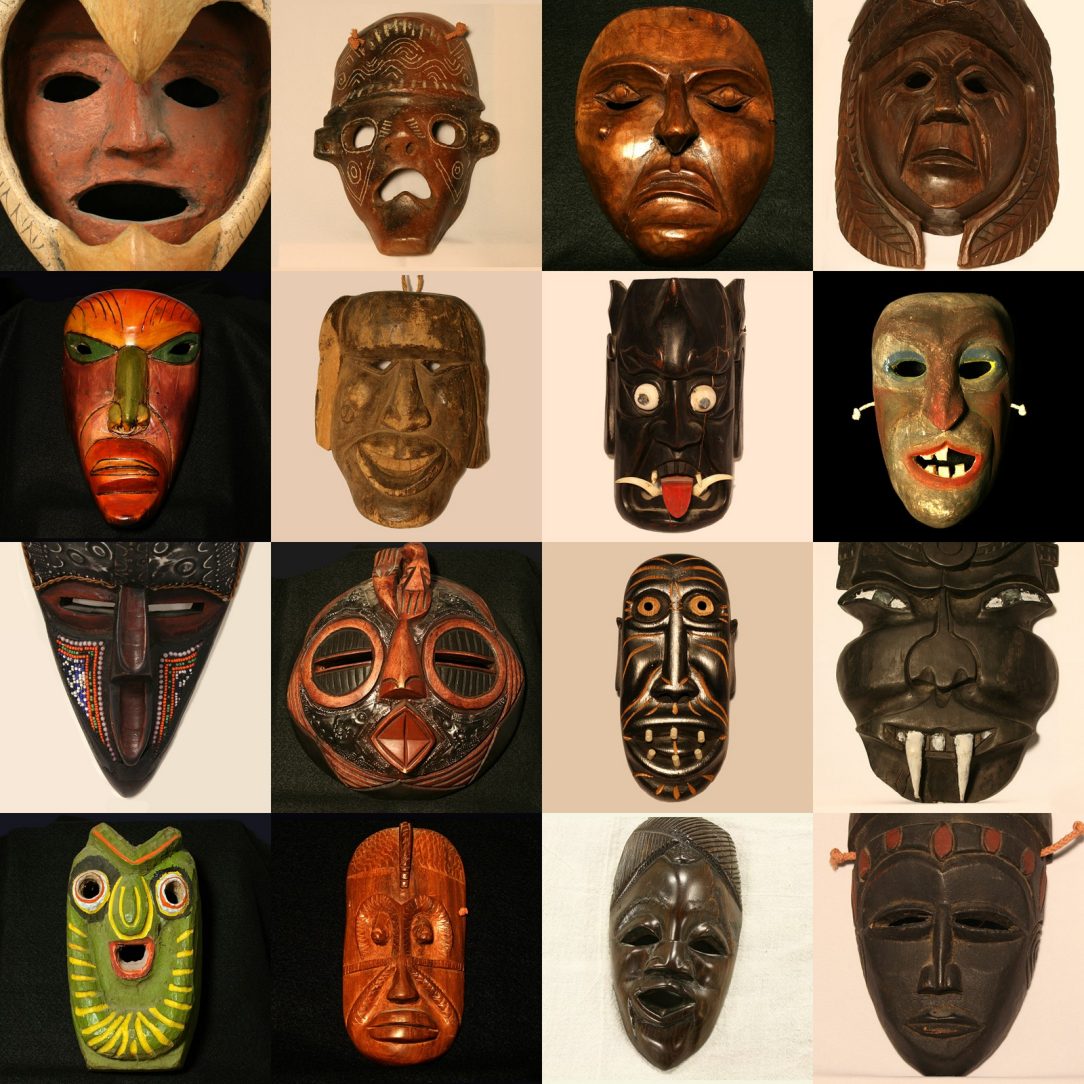
Photographs of masks 17–32 used in the current study. The masks are arranged numerically from top-left (Masks 17 & 18) to bottom-right (Masks 31 & 32). These photographs of a subset of the David Coffey collection are reproduced courtesy of the Kentucky Museum, located on the campus of Western Kentucky University (the photographer was the first author, J. Farley Norman).

The visual stimuli were photographs of 32 masks (the camera used was a Canon Rebel XTi) taken from a larger set of 140 that were originally collected by the late Dr. David M. Coffey and are now housed at the Kentucky Museum, located on the campus of Western Kentucky University. These 32 masks (see Figs 2 and 3) were chosen in order to satisfy two criteria. One was to create a stimulus set that reflects the work of a wide variety of countries and cultures; the stimulus set included six masks from Central America, six masks from South America, five masks from Africa and the Comoros Islands, three masks from North America, three Masks from Asia, and two masks from Atlantic Ocean islands (the country of origin of 7 masks was undocumented). A second criterion was to include masks that incorporate a wide variety of actions according to the Ekman Facial Action Coding System [12]. For example, Mask 5 expresses action units 26 (Jaw Drop) and 2 (Outer Brow Raiser), while Mask 21 expresses action units 24 (Lip Pressor), 9 (Nose Wrinkler), and 4 (Brow Lowerer). Some of the masks were photographed against dark backgrounds and some were photographed against light backgrounds, whichever background served to give the best contrast and visibility to each mask and its constituent features. All of our masks when viewed against their backgrounds possessed a Michelson contrast [13] that was 0.9 or higher (mean contrast = 0.95, sd = 0.02; the individual luminances of the background and adjacent interior parts of the mask that differed most from the background were measured using a PMLX photometer with a fiber optic PM10 probe [Quantum Instruments Inc., Hauppauge, NY]). Each of the full-color stimulus images had a resolution of 800 x 800 pixels and subtended a visual angle of approximately 17 degrees.

The stimulus presentations and the collection of participant responses was performed by an Apple Mac Pro computer (Dual Quad-Core processors, with ATI Radeon HD 5770 hardware-accelerated graphics) using an Apple 27-inch LED Cinema Display. The monitor was located at a 60 cm viewing distance from the participant.

### Procedure

For each mask, the participants were asked to numerically rate the perceived amount of the six basic emotions common across cultures [14–17]: happiness, sadness, anger, fear, surprise, and disgust. In addition to analyzing the participants’ individual ratings of the six basic emotions for each mask, we also calculated an overall measure of perceived emotional intensity. The overall magnitude (intensity) of such a six-dimensional response (i.e., vector) was calculated in the conventional way (e.g., Marsden & Tromba [18], pp. 38–39) and was defined here as:

$$\mathrm{intensity}=\sqrt{{happiness}^{2}+{sadness}^{2}+{anger}^{2}+{fear}^{2}+{surprise}^{2}+{disgust}^{2}}$$ (1)

The participants made their judgments for each of the six emotions by adjusting sliders (with the computer’s mouse) along continuous scales which varied from zero (none of that emotion perceived) to 100 (maximal expressed happiness, maximal expressed sadness, etc.). Graphical response scales with a resolution of up to 100 have commonly been used for almost a century (Guilford [19], p. 270); the sliders used in the current study were similar to those used in our laboratory in previous research [20–21]. Each participant was presented with the 32 masks (Figs 2 and 3) successively in a completely random order. The participants were allowed as much time as they wished to evaluate the stimulus masks. The participants were also told to verbally indicate any emotions they perceived, if they were different from the basic six.

### Participants

The participants were 51 young adults (mean age = 22.2 years, sd = 5.0). All gave written consent prior to participation in the experiment. The experiment was approved by the Western Kentucky University Institutional Review Board. Our research was carried out in accordance with the Code of Ethics of the World Medical Association (Declaration of Helsinki). All participants were naïve regarding the purposes of the experiment. The visual acuity of the participants was good: the acuity measured at 1 meter (using a Precision Vision 2195 eye chart) was -0.03 LogMAR (log minimum angle of resolution). Zero LogMAR indicates normal levels of visual acuity, while negative and positive values indicate better than normal acuity and worse than normal acuity, respectively.

## Results

**Fig 4:**
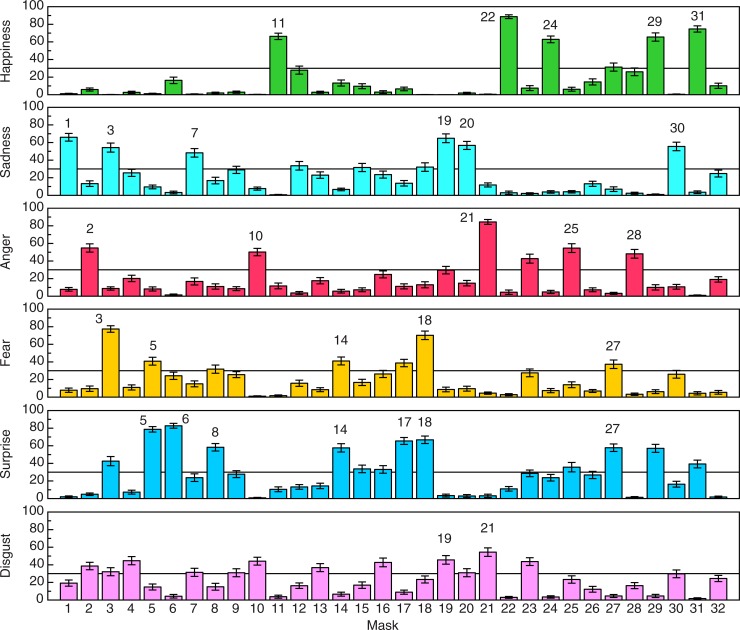
The participants’ ratings of perceived emotion are plotted for each of the six basic emotions common across cultures [12]: Happiness, sadness, anger, fear, surprise, and disgust. It is important to note that only a small minority of the masks were perceived as being strongly emotional for any particular emotion. Few masks, for example, produce ratings above 30 percent (solid line) of maximum possible perceived happiness, sadness, anger, fear, surprise, and disgust. Thirty percent was chosen, because it lies near the 75th percentile point (31.6) of the total distribution of 192 ratings shown in the figure. Thus, only approximately 25 percent of all perceived emotion magnitudes rise above a value of 30. The error bars indicate ± 1 SE.

The overall participant results are shown in Fig 4, which plots perceived happiness, sadness, anger, fear, surprise, and disgust for each of the 32 stimulus masks. One can readily see that there are wide variations in perceived emotion across the 32 masks. It is clear that some masks produce intense perceptions of single emotions (e.g., Mask 22 for happiness, Mask 1 for sadness, Mask 25 for anger, Mask 6 for surprise, Mask 4 for disgust). These variations in perceived emotion were verified as being significant (using within-subjects analyses of variance) for all six of the basic emotions identified by Ekman et al. [15]: Happiness (F(31, 1550) = 107.3, p < .000001, η_p_^2^ = .68), sadness (F(31, 1550) = 42.6, p < .000001, η_p_^2^ = .46), anger (F(31, 1550) = 47.0, p < .000001, η_p_^2^ = .49), fear (F(31, 1550) = 41.9, p < .000001, η_p_^2^ = .46), surprise (F(31, 1550) = 61.9, p < .000001, η_p_^2^ = .55), and disgust (F(31, 1550) = 22.7, p < .000001, η_p_^2^ = .31).

Some masks produced only intense perceptions of happiness with no other significant accompanying emotion (e.g., masks 11 & 22); other masks produced intense single perceptions of sadness, anger, surprise, and disgust. Such masks (producing perceptions of only a single emotion) did not exist for fear within our stimulus set. Instead, intense perceptions of fear were always accompanied by significant perceptions of other emotions. For example, Mask 3 was perceived as being intensely fearful, but was also rated highly for sadness, surprise, and disgust (see Fig 4). Masks 10 and 21 were rated highly for anger, but also for disgust. Other blends of emotion [22] were also observed: a) Happiness and surprise (masks 29 & 31), b) Anger and surprise (mask 25), c) Fear and Surprise (masks 5, 8, 14, & 17), d) Sadness and disgust (masks 7, 19, & 20), e) Sadness and surprise (mask 15), f) Surprise and disgust (mask 16), g) Sadness, fear, and surprise (mask 18), and h) Happiness, fear, and surprise (mask 27).

**Fig 5:**
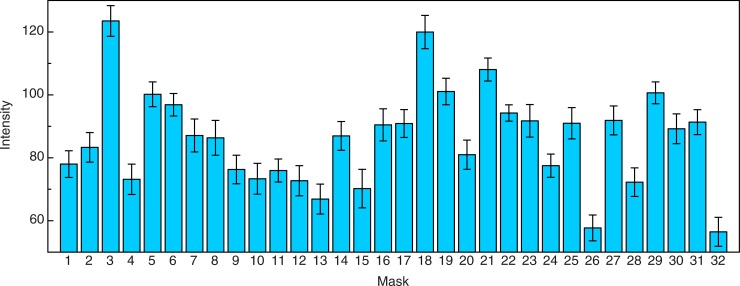
Experimental results. The overall perceived emotional intensity of each mask. The mask that produced the most intense perception of emotion was Mask 3, while the mask that produced the least intense perception of emotion was Mask 32. The error bars indicate ± 1 SE.

The participants’ perceptions of overall emotional intensity are shown in Fig 5. It is readily apparent that the facial expressions of masks 3 and 18 were perceived as most intense, while those of masks 26 and 32 were perceived as least intense (i.e., most neutral). Both masks 3 and 18 produce perceptions of strong fear (which Ekman & Friesen [22] refer to as “terror”), as well as substantial amounts of sadness, surprise, and disgust. It is also interesting that mask 26, one of the relatively neutral masks, while it does possess eyes, nose, and mouth, does not appear human (one of our participants said that this mask looks like a bird; notice the owl-like eyes and the beak-like mouth). Perhaps this nonhuman (bird-like) appearance accounts for the fact that our participants perceived relatively little emotion from mask 26. Given the wide variation in perceived emotional intensity across the 32 masks, it is not surprising that the effect of mask upon intensity was statistically significant (F(31, 1550) = 21.3, p < .000001, η_p_^2^ = .30).

With regards to the qualitative (i.e., verbal) results, only eight participants out of 51 (15.7 percent) perceived an emotion other than the basic six that Ekman et al. [15] described. Only for two masks was there any commonality in verbal description. Mask 7 was described by two participants as being “sleepy”, while Mask 28 was described as having “evil intent” (malice, cunning, etc.).

## Discussion

Twenty years ago, two studies [10–11] evaluated participants’ perceptions of emotion when viewing single Japanese Noh masks. These studies only evaluated happiness, sadness, and surprise, and did not evaluate other fundamental emotions, such as anger, fear, and disgust. In addition, these previous studies [10–11], despite their pioneering nature, only required participants to make simple binary choices about their mask stimuli (for example, to indicate whether a particular image was perceived as “happy” or “sad”). The current study, therefore, represents the first ever scientific investigation of emotion perception that 1) investigated many masks (32) from a wide variety of cultures, 2) required participants to evaluate the actual magnitude of the emotions they perceived, and 3) studied all six of the emotions common to cultures across the world (happiness, sadness, anger, fear, surprise, and disgust). It is true that the participants in our current study were all younger adults (mean age was 22.2 years). A very recent study (Mienaltowski, Groh, Hahn, & Norman [23]) found that older adults (mean age was 70.7 years) were equally good as younger adults at detecting high-intensity expressions of anger and fear, but they were nevertheless less sensitive at detecting low-intensity expressions of fear. Given this finding, it would be worthwhile for future investigations of emotion perception from masks to include judgments from older adults.

Why did the masks employed in this study produce such strong perceptions of emotion (see Figs 4 and 5)? After all, they are inanimate objects, created by artists out of wood, stone, ceramic, and metal; thus, the masks themselves are not emotional. We, the perceivers, nevertheless experience emotions when we view the masks. The simple answer is that we perceive emotion, because the artists who created these masks included facial features that mimic those that are produced by us in everyday life. Consider first of all Mask 5; our participants perceived high amounts of surprise (and some fear). According to Darwin [14] and Ekman and Friesen [22], surprise is indicated in a human face when 1) the eyebrows are raised, 2) the eyes are opened wide, and 3) the jaw is dropped so that the lips part. As can be seen from Fig 2, all of these elements of human surprise and facial expression are present and clearly evident in mask 5. As a second example, consider Mask 21, which for our participants was the highest-rated mask for both anger and disgust. The narrow penetrating eyes (compare to Fig 33A of Ekman & Friesen [22]) and lips pressed tightly together (compare to Figs 34A and 34B of Ekman & Friesen [22]) indicate anger, while disgust is indicated by a highly visible naso-labial fold and a “wrinkled nose” (Ekman & Friesen [22], p. 71). As final examples, consider Masks 1 and 19, which were rated by our participants as expressing the most sadness. Mask 1 is perceived to be sad, because the eyes droop and are cast downwards, while Mask 19’s sadness occurs primarily because the corners of the mouth are drawn downwards [14, 22].

Charles Darwin [14] noted (p. 360) that artists find it very difficult to “depict the characteristic signs of each particular state of mind”. This is undoubtedly so, because many masks do not produce effective perceptions of emotion in human observers (notice that even within our stimulus set, Masks 26 and 32 were perceived as being almost completely neutral). Despite the difficulties, many talented artists worldwide have learned the reliable facial features that express human emotion [14, 17, 22] and have recreated them in stone, wood, ceramic, and metal for ceremonial, judicial, recreational, and other cultural purposes. In our opinion, the mask illustrated in Fig 1 is an excellent example of an exquisite piece of art (Pacific Northwest of North America) that has the power to produce effective and compelling perceptions of emotion in human perceivers.

## Supporting information

S1 File Individual participant estimates of happiness. (XLSX)

S2 File Individual participant estimates of sadness. (XLSX)

S3 File Individual participant estimates of anger. (XLSX)

S4 File Individual participant estimates of fear. (XLSX)

S5 File Individual participant estimates of surprise. (XLSX)

S6 File Individual participant estimates of disgust. (XLSX)
